# Peritoneal-Membrane Characteristics and Hypervolemia Management in Peritoneal Dialysis: A Randomized Control Trial

**DOI:** 10.3390/membranes11100768

**Published:** 2021-10-08

**Authors:** Szu-Yuan Li, Chiao-Lin Chuang, Chih-Ching Lin, Shin-Hung Tsai, Jinn-Yang Chen

**Affiliations:** 1 Division of Nephrology, Department of Medicine, Taipei Veterans General Hospital and School of Medicine, National Yang-Ming Chiao-Tung University, Taipei 112201, Taiwan; syli@vghtpe.gov.tw (S.-Y.L.); lincc2@vghtpe.gov.tw (C.-C.L.); 2 Division of General Medicine, Department of Medicine, Taipei Veterans General Hospital and School of Medicine, National Yang-Ming Chiao-Tung University, Taipei 112201, Taiwan; clchuang@vghtpe.gov.tw; 3 Division of Nephrology, Department of Medicine, Cheng Hsin General Hospital, Taipei 112201, Taiwan; ch6583@chgh.org.tw

**Keywords:** peritoneal dialysis, blood pressure, residual renal funciton, bioimpedance, peritoneal membrane

## Abstract

Excessive bodily-fluid retention is the major cause of hypertension and congestive heart failure in patients with end-stage renal disease. Compared to hemodialysis, peritoneal dialysis (PD) uses the abdominal peritoneum as a semipermeable dialysis membrane, providing continuous therapy as natural kidneys, and having fewer hemodynamic changes. One major challenge of PD treatment is to determine the dry weight, especially considering that the speed of small solutes and fluid across the peritoneal membrane varies among individuals; considerable between-patient variability is expected in both solute transportation and ultrafiltration capacity. This study explores the influence of peritoneal-membrane characteristics in the hydration status in patients on PD. A randomized control trial compares the bioimpedance-assessed dry weight with clinical judgment alone. A high peritoneal membrane D/P ratio was associated with the extracellular/total body water ratio, dialysate protein loss, and poor nutritional status in patients on PD. After a six-month intervention, patients with monthly bioimpedance analysis (BIA) assistance had better fluid (−1.2 ± 0.4 vs. 0.1 ± 0.4 kg, *p* = 0.014) and blood-pressure (124.7 ± 2.7 vs. 136.8 ± 2.8 mmHg, *p* < 0.001) control; however, hydration status and blood pressure returned to the baseline after we prolonged BIA assistance to a 3-month interval. The dry-weight reduction process had no negative effect on residual renal function or peritoneal-membrane function. We concluded that peritoneal-membrane characteristics affect fluid and nutritional status in patients on PD, and BIA is a helpful objective technique for fluid assessment for PD.

## 1. Introduction

The pathogenesis of hypertension (HTN) and congestive heart failure in patients with end-stage renal disease (ESRD) is multifactorial, and fluid overload is one of the most important determining factors [1,2,3,4]. Patients with ESRD experience expanded circulating volume due to excess fluid and sodium retention. In addition to HTN, fluid overload induces congestive heart failure in patients with ESRD [3,4,5,6,7]. An artificial dialysis membrane is used to remove body toxins and fluid in hemodialysis, and the patient’s dry weight is defined as the lowest postdialysis body weight without hypotension. This weight should be similar to what a person with normal kidney function would weigh after urinating. There is no standard measure of dry weight, so it is difficult to ascertain the adequacy of fluid removal for individual patients [8]. Several different techniques, including inferior diameter, natriuretic peptide, and bioimpedance, were used to derive a more standard method of assessing dry weight [9,10,11,12]; however, no single method has emerged as a gold standard, so nephrologists need to frequently adjust the patient’s dry weight on the basis of interdialytic weight gain and intradialytic hemodynamic changes [13]. In addition, the abdominal peritoneum is used as a semipermeable dialysis membrane in peritoneal dialysis (PD) to provide 24 h of continuous toxin and fluid removal. Patients’ dry-weight assessment is more difficult due to the steady hemodynamical status and body weight. Another key factor that, dry-weight setting challenging in PD is that the speed of small solute diffusion across the peritoneal membrane varies among individuals; the same dialysate prescription can result in different ultrafiltration rates among patients. Nephrologists use the peritoneal equilibration test (PET) to categorize peritoneal-membrane characteristics in four groups, but whether peritoneal-membrane characteristics influence hydration status in patients on PD is controversial. Another concern of dry-weight setting in PD is the risk of damaging residual renal function (RRF) because rapid bodily-fluid reduction leads to urine volume depletion and RRF loss in patients on PD [6]. Given the strong correlation of RRF and survival in PD [14], nephrologists face the dilemma of better blood-pressure (BP) control or renal function maintenance. As a consequence, a considerable portion of patients on PD have HTN [15,16].

A careful physical examination is considered to be the gold standard to assess dry body weight in PD; an objective measurement would be a useful addition, especially for patients who are free from edema but need antihypertensive drugs to control BP. By using bioimpedance analysis (BIA), several groups found that most patients on PD have excess total body water (TBW) and extracellular water (ECW), and the overhydration status is comparable or even greater than the values found in patients on HD before a dialysis session [17,18,19]. In the current study, the effect of peritoneal-membrane characteristics in hydration status was studied, and a one-year randomized control trial using BIA to correct overhydration was conducted.

## 2. Methods

### 2.1. Study Design

Patients with stable PD (who had received PD treatment for >3 months), and age- and sex-matched healthy subjects received a baseline BIA measurement of bodily-fluid composition. A total of 122 prevalent patients with stable PD in Taipei Veterans General Hospital and Cheng Hsin General Hospital Taiwan were recruited. Exclusion criteria were patients with severe congestive heart failure (defined as New York Heart Association functional class IV), unstable angina, or malignancy. Patients who wore heart pacemakers or intracardiac defibrillators were excluded due to the adverse effect of bioimpedance measurement on pacemaker function. Apart from the exclusion criteria, no selection criteria were applied. Each patient gave written informed consent before study participation. All patients on PD received a standard 4 h PET to measure peritoneal membrane function, and analyze the influence of peritoneal membrane characteristics in bodily-fluid composition and nutritional status. After the baseline PET and BIA measurement, patients were randomized into intervention and control groups, and their dry weight was targeted by BIA assistance or clinical judgement only, respectively. The study design was approved by Taipei Veterans General Hospital and Cheng Hsin General Hospital clinical trial ethics committees.

### 2.2. Peritoneal-Membrane Equilibration Analysis

The PET is a semiquantitative assessment of peritoneal-membrane transport function in patients in PD [20]. The solute transport rates across the peritoneal membrane were assessed by their equilibration rates between peritoneal capillary blood and dialysate. The ratio of solute concentration in dialysate and plasma (D/P ratio) at various time points during the dwell signifies the extent of solute equilibration. This ratio is determined by creatinine that is transported from the capillary blood to the dialysate, the standard fixed fill volume of 2 L of a 2.27% glucose solution was used over a 4 h dwell to characterize the rate of transfer of solute and water across the peritoneal membrane [21,22].

### 2.3. Intervention

After giving their informed consent, patients on PD were randomly assigned to the study or control group. In the intervention group, patients received BIA measurements every month to assess their hydration status. If the height-normalized ECW (nECW) was higher than the target level (defined as nECW above the 90th percentile of normotensive patients on PD), body weight decreases by a rate of 0.5 kg per month. A registered dietitian nutritionist developed and implemented low-salt diet programs for our patients. In the study period, the registered dietitian nutritionist continuously assessed patient understanding and compliance with nutritional intervention, and body-weight reduction was achieved by salt restriction of 5 g/day in combination with 120 mg/day furosemide in patients with urine volume >200 mL/day. If the nECW was still higher than the target level, ultrafiltration was enhanced by either hypertonic PD solution or icodextrin. Not all patients were entitled to use icodextrin due to the reimbursement policy in Taiwan’s National Health Insurance, but four patients (two in each group) received a 4.5% hypertonic PD solution. Body-weight reduction was stopped if RRF had decreased by 30% from the baseline, postural hypotension was present, or nECW had achieved the target level. In the control group, a dry-weight setting was based on the clinical judgment of two independent nephrologists, with identical body-weight reduction strategies with the study group. During the study period, antihypertensive drug dosage was accordingly adjusted to each patient’s clinical condition. After 6 months of the intervention period, BIA measurement shifted to a 3-month interval in the study group. In the control group, patients only had BIA measurement 3 times, namely, at the beginning of the study, and on the 6th and 12th months.

BP used in this study was assessed by the mean values of home BP on the 1st, 10th, and 20th day every month. Patient were categorized as normotensive (mean blood pressure less than 140/90 mmHg that did not require anti-HTN medication), hypertensive (taking HTN medication or mean blood pressure greater 140/90 mmHg), and poorly controlled hypertension (mean blood pressure greater than 160/100 mmHg). RRF was assessed by 24 h urine collection and monthly blood sampling. Overhydration is linked to malnutrition and inflammation in patients with ESRD [23,24]; thus, serum albumin and highly sensitive CRP were collected at 0, 6th, and 12th months. Blood and urine samples were measured using a Hitachi 7600 chemical autoanalyzer (Hitachi. Co., Ltd., Tokyo, Japan) in a CAP-qualified central laboratory.

### 2.4. Body Composition Analysis

Body composition analysis was performed by a tetrapolar 8-point tactile electrode, standing type multifrequency segmental bioimpedance analyzer (InBody 720, Biospace, Korea), which offered accurate estimates of total and appendicular body composition [25]. Patients had to empty the urinary bladder, drain the PD solution out, and wear light clothing before measurement for accurate measurement readings. Extracellular water (ECW), intracellular water (ICW), and TBW were determined by the analyzer of bioelectrical impedance spectroscopy [26].

### 2.5. Statistical Analyses

Data were expressed as mean ± SD. Differences between the study and control groups were analyzed by unpaired Student’s t-test. A nonparametric test was used for data that were not normally distributed (antihypertensive drug dosage, RRF, and high-sensitivity C-reactive protein (HS-CRP)). Correlations between continuous variables were estimated by Pearson correlations. We used two-way repeated-measures ANOVA to determine if the two groups diverged BP and residual renal function over the study period. The Shapiro–Wilk and Mauchly’s sphericity tests were used to test the normal distribution of the data, and validate the potential bias of repeat measurement in different time points. All probabilities were two-tailed examined. A *p* value < 0.05 was considered to be statistically significant. In regard to sample-size calculation, according to the initial blood-pressure variance among all participants, we needed 40 patients in each group to a 95% confidence interval with an 80% power significant 10 mmHg blood-pressure difference.

## 3. Results

### 3.1. Peritoneal-Membrane Characteristics and Hydration Index

**Figure 1:**
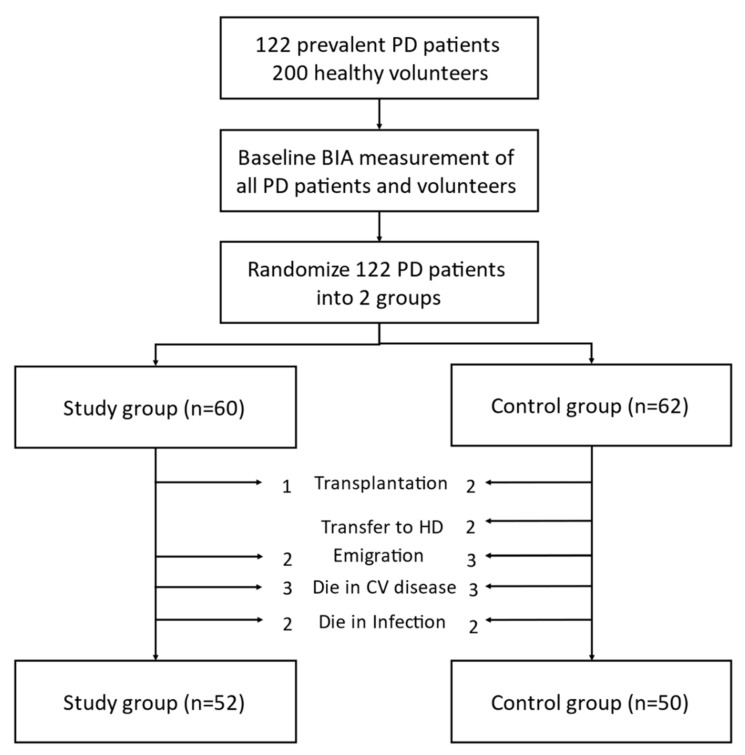
Study design. Consort diagram showing number of patients recruited into the study and dropout reasons.

**Figure 2:**
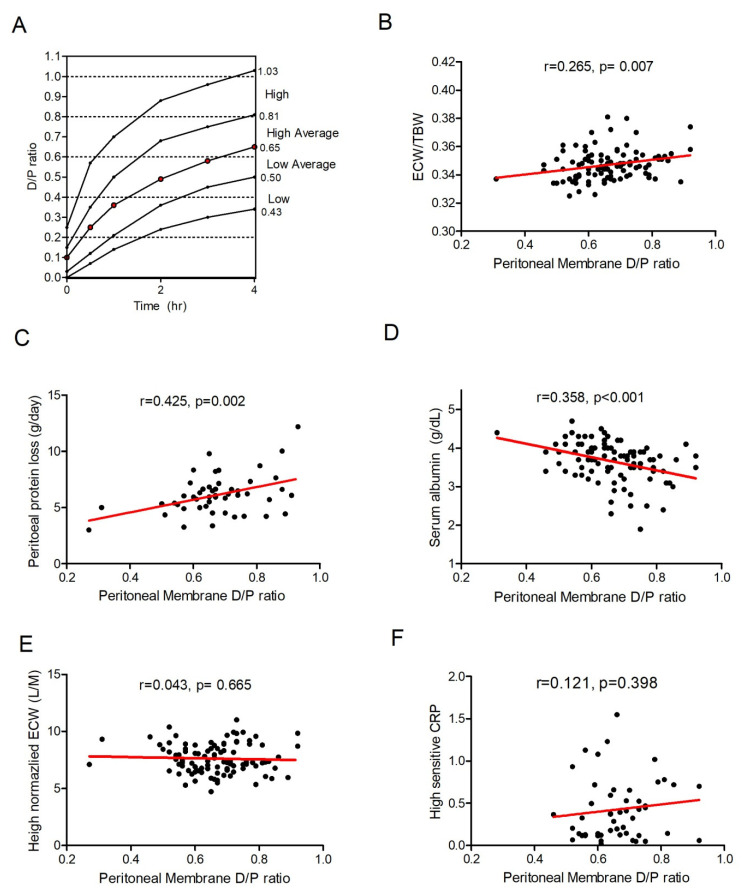
Peritoneal-membrane characteristics associated with bodily-fluid composition ECW/TBW and protein malnutrition. (**A**) Standard PET categorized peritoneal-membrane characteristics into four groups on the basis of the speed of creatine diffusion from the capillary into the dialysate. (**B**) Association of peritoneal D/P ratio to ECW/TBW, (**C**) dialysate protein loss, (**D**) serum albumin, (**E**) nECW, and (**F**) HS-CRP.

Among the 120 recruited patients on stable PD, 102 patients finished a 1-year study. Patient flow is summarized in Figure 1. Baseline peritoneal membrane D/P ratio was positively associated with ECW/TBW ratio (r = 0.256, *p* = 0.007) and peritoneal protein loss (r = 0.425, *p* = 0.002), and negatively associated with serum albumin (r = −0.358, *p* < 0.001). Our data revealed that ECW/TBW is strongly associated with serum albumin, and suggested a nutritional parameter rather than a hydration index in PD; therefore, we used nECW in the following steps, as previously suggested [27,28,29], which was not influenced by peritoneal-membrane characteristics or serum albumin in our study. Association between peritoneal membrane D/P ratio and inflammation marker HS-CRP was not found. Results are summarized in Figure 2.

### 3.2. Most Patients on PD Are Fluid-Overloaded

**Figure 3:**
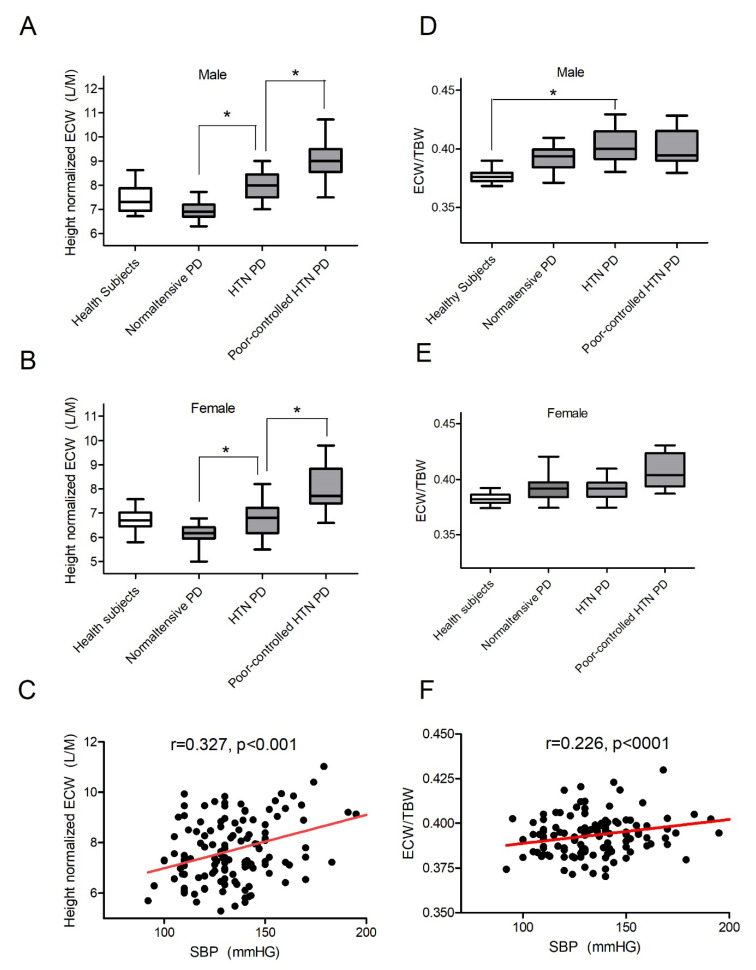
Fluid status in PD patients and healthy participants. Height-normalized ECW in different blood-pressure groups in (**A**) males and (**B**) females. (**C**) Association of nECW and BP. Hydration status also illustrated in ECW/TBW in (**D**) males and (**E**) females. (**F**) Association of ECW and BP. Hydration index presented as box plot. Box indicates 25th–75th percentile range, and capped bars indicate the 10th–90th percentile range. Line across the box indicates medium. Between-group difference, * *p* < 0.05.

Among the 122 PD patients, 35 were normotensive, 67 were hypertensive, and 20 were poorly controlled hypertensive. Baseline screening showed that nECW was positively correlated to age (r = 0.184, *p* = 0.036), systolic BP (SBP) (r = 0.327, *p* = 0.001), residual urine volume (r = 0.239, *p* = 0.015), and renal function nWCC (r = 0.303, *p* = 0.002). The value was not correlated to peritoneal membrane D/P ratio, dialysis adequacy serum albumin, HS-CRP, or renal nKT/V. In females, the nECW was 6.09 ± 0.49 L/m in normotension vs. 6.88 ± 0.75 L/m (*p* < 0.001) in drug-controlled HTN vs. 7.60 ± 0.73 L/m (*p* < 0.001) in patients on PD with poorly controlled HTN. In males, the nECW was 6.92 ± 0.39 L/m in normotension vs. 8.24 ± 0.61 L/m (*p* = 0.003) in drug-controlled HTN vs. 9.09 ± 1.18 L/m (*p* < 0.001) in patients on PD with poor controlled hypertensive. In 200 healthy sex- and age-matched participants, BIA analysis revealed that females had less nECW than males did, and height nECW was 6.89 ± 0.56 and 7.66 ± 0.79 L/m, respectively. ECW/TBW ratio was also positively associated with blood pressure, but the magnitude of the association was weaker. In summary, these data suggest that overhydration is a common problem among PD patients, and nECW may be a better hydration index than the ECW/TBW ratio is in this population. Results are summarized in Table 1 and Figure 3.

### 3.3. Normalized Overhydration Improves BP Control in PD

No differences were found in the peritoneal-membrane D/P ratio, body weight, BP, anti-HTN drug dosage, serum albumin, and RRF between the study and control groups before intervention. Initial demographic data are summarized in Table 2. In a six-month intervention period, the study group experienced a 1.2 kg body-weight reduction, whereas the control group gained 0.1 kg (*p* = 0.014). The nECW value also reached significant difference (−0.41 ± 0.13 L/m, *p* = 0.04). The ECW/TBW ratio did not reflect these changes. The study group ended up with a better systolic (124.7 vs. 136.8 mmHg, *p* < 0.001) and diastolic (74.9 vs. 80.5 mmHg, *p* = 0.05) BP control. The reduction in SBP was positively associated with nECW changes (r = 0.397, *p* = 0.007). At the end of the intervention period, serum albumin was comparable in the study (3.77 ± 0.08 g/dL) and control (3.57 ± 0.07 g/dL) groups (*p* = 0.105). No difference was found in serum HS-CRP level (0.520 ± 0.114 vs. 0.620 ± 0.093, *p* = 0.551). Delta nECW was not correlated to HS-CRP or albumin change.

**Figure 4:**
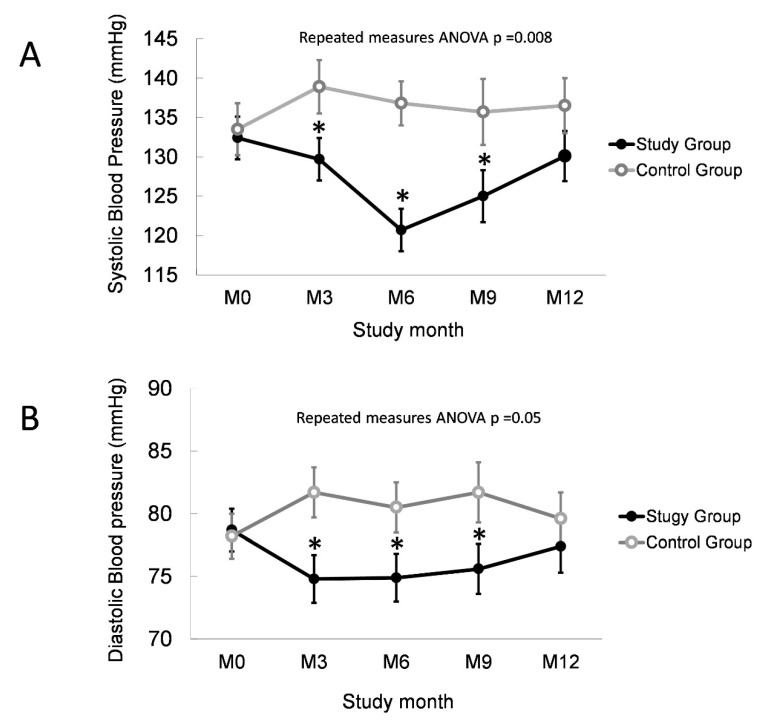
Serial blood-pressure and body-weight changes. Serial (**A**) systolic and (**B**) diastolic blood pressure in two groups. Between-group difference, * *p* < 0.05.

In the maintenance phase, the SBP of the two groups became 127 vs. 135 mmHg (*p* = 0.045) in the 9th month, and insignificant at the end of the study (*p* = NS). BIA measurement in the 12th month also revealed that nECW had returned to the baseline. These results suggested that most patients on PD are insidious-fluid-overloaded, so normalized overhydration can correct HTN in patients on PD; however, maintaining true long-term euvolemic status without the assistance of an objective technique is difficult. These results are summarized in Figure 4. Serial clinical parameters are summarized in Table 3.

### 3.4. Normalized Overhydration Does Not Damage RRF or Peritoneal Membrane Function

**Figure 5:**
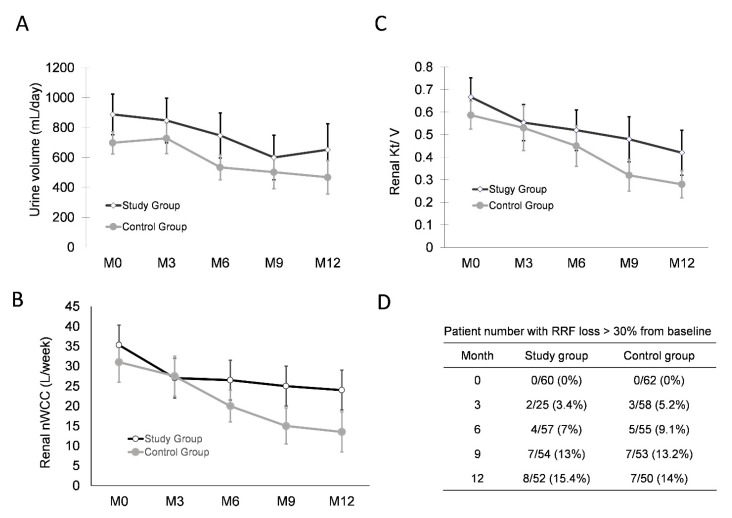
Serial changes of residual renal function. In the whole study period, both groups had RRF reduction, but there was no intergroup difference between the two groups. Renal function expressed in (**A**) urine volume, (**B**) renal nWCC, and (**C**) renal nKT/V. (**D**) Number and percentage of individuals with RRF > 30% during study period.

The decline in renal function does not stop after the initiation of renal replacement therapy. Patients on HD lose their RRF much sooner than patients on PD do because of the rapid hemodynamic changes in hemodialysis sessions three times a week. In the current study, significant RRF loss occurred over a 1-year study period in both groups, but without intergroup difference. Urine volume and toxin removal amount were separately quantified to measure the RRF. No statistically significant difference was found in urine volume between the two groups in the beginning (886 ± 136 vs. 698 ± 75 mL, *p* = 0.226), the end of intervention (747 ± 150 vs. 534 ± 84 mL, *p* = 0.224), and the end of the study (562 ± 174 vs. 468 ± 112 mL, *p* = 0.368). Two different methods were used to quantify residual renal toxin clearance, namely, renal nWCC and nKT/V. Like urine volume, renal toxin clearance decreased over time, but without intergroup difference between the two groups in the whole study period. Results are summarized in Figure 5. Peritoneal-membrane function was followed up in a 6-month interval, which revealed no significant intergroup changes during the intervention and maintenance period.

## 4. Discussion

Many patients with ESRD are globally treated with dialysis. PD uses the peritoneal membrane as a semipermeable membrane for solute transfer and ultrafiltration. The properties of this membrane are important determinants for selecting the optimal treatment regimen, but vary among individuals. PET was developed some 25 years ago and has been used to help prescribe PD. Our study revealed that peritoneal-membrane characteristics both influence intrabdominal solute diffusion efficiency, and contribute to the total bodily fluid distribution of ECW/TBW among patients on PD. Since D/P ratio is not associated with HS-CRP, the altered ECW/TBW ratio is probably not caused by systemic inflammation.

Several techniques have been proposed and investigated to measure body composition in clinical practice. Among these, BIA has attracted much attention due to its practicality in fluid status assessment in patients in PD [30,31,32,33,34]. With multifrequency, BIA, ECW, ICW, and TBW can be conveniently estimated in a single measurement. One major problem involving the utilization of BIA results as hydration index is that normalization reference for ECW could influence hydration status classification [28]. In our work, many factors influenced the ECW/TBW ratio in patients on PD, including peritoneal membrane D/P ratio, serum albumin, and residual urine volume. Several other groups also found a similar bias of ECW/TBW as a hydration index in PD [31,35,36,37,38]. In hemodialysis, ECW reduction is abrupt, and the ECW/TBW ratio decreased after a 4 h HD session [17]; thus, ECW/TBW may be used as a hydration index in HD [39]. However, TBW changed in the same proportion as that of ECW during a hypervolemia-correction process, making the ECW/TBW an inappropriate reference for hydration index in PD due to its slow and continuous fluid status change. Considering the strong influence of serum albumin on ECW/TBW ratio, ECW/TBW is a better nutrition marker than hydration index in PD is [35,37].

In our study, patients on PD with normotension had a lower nECW than that of healthy participants. Van de Kerkhof et al. [28], and Wang et al. [34] also made similar observations. This result is contradictory to the concept that most patients on PD are overhydrated. In addition to hypervolemia, numerous factors rendered patients with uremia to HTN, for example, chronic inflammation [40], sympathetic nerve system overactivation [41], and arterial stiffness [42]. On the basis of these HTN-predisposing factors, patients on PD are not required to have a lower nECW than that of healthy participants to achieve normotension. In our study, the value of height nECW was much lower than the suggested reference value of normovolemia, speculated as 10.96 L/m in males and 9.13 L/m in females on PD in a cross-sectional observational study [28]. The most reasonable explanation is that the BIA machine used in a previous study (Xitron BIS 4000, San Diego, Calif, USA) overestimated the ECW. The ECW/TBW ratio in healthy participants was 55%, a much higher value than the generally accepted ECW that contributed to one-third of TBW. The Xition BIA overestimated the ECW of 2.7 L in patients on PD [30]. Factoring the 2.7 L difference, the reference nECW values would be similar in these two studies. In our work, the mean ratio of ECW/TBW in healthy participants was 33.4%, which fit the proportion of bodily-fluid distribution.

The ideal sodium intake and fluid control in patients with PD are long-standing challenges for nephrologists. Our data showed that the blood pressure and hydration status of the intervention group improved at the end of intervention, but there was no difference of intergroup PD dialysate utilization, suggesting that the reduction in salt intake could be the reason. Guanl et al. reported a 2 kg body-weight reduction within 4 weeks by strict sodium and fluid restriction alone, accompanied with improved BP control and left ventricular hypertrophy (LVH) regression. In that study, the addition of hypertonic PD fluid rendered patients to lose 4 kg to achieve normotension if salt restriction alone failed to normalize BP; the patients’ fluid status was not addressed in detail, but a considerable number of their patients may have suffered from dehydration. Therefore this impressive improvement in BP was offset by a loss of residual urine volume [6]. Two randomized control studies regarding the effect of icodextrin in PD may inform nephrologists on the relationship between fluid status and RRF. The use of icodextrin increased the ultrafiltration amount in both studies; however, the effect of icodextrin on RRF was contradictory [43,44]. One study compared the effect of icodextrin and 1.36% glucose PD dialysate. ECW decreased by 2.1 L in the icodextrin-treated group, as 1.7 kg was lost within 4 months, but this group also had a significant loss of RRF [43]. Another trial compared icodextrin versus 2.27% glucose PD dialysate, which revealed that the icodextrin-treated group had a mean ECW of 1 L reduction in 6 months, and better maintenance of RRF was observed [44]. The reason behind the difference in RRF between these two studies is unclear; however, Konings et al. assumed that underhydration during the use of icodextrin would explain the decline in RRF in their study. This is supported by excluding a dehydrated patient, and the decline in RRF between the study and control group was comparable [45].

In the current study, a practical method of dry-weight assessment in PD was demonstrated. A combination of multifrequency BIA examination, salt restriction, diuretic agent, and increase ultrafiltration improved fluid status and BP in most patients on PD. In the intervention period, hydration status in the study group was objectively assessed on a monthly basis; body-weight reduction was gradual and step-by-step, resulting in better BP control without compromising the RRF. In the maintenance phase, fluid status was measured in a 3-month interval; such a relaxed approach towards fluid control diminished. In this study, any patient suffering a loss of 30% RRF from the baseline stopped the process of dry-weight reduction. Nevertheless, such a measure did not slow down the rate of decline in RRF in the majority of patients. Most PD patients presented abrupt RRF loss in this study, are caused by infection, ischemic heart disease, or side effects other than severe dehydration. In the intervention period, the rate of decline in urine volume and RRF was comparable between the study and control groups, which gives us confidence that fluid overload correction does not impair RRF in patients on PD if dehydration is prevented. It may not be practical to set a rigid target value for all patients on PD, but the incorporation of BIA into clinical judgment is useful for longitudinal fluid status monitoring.

Our study has several limitations. First, echocardiography was not performed in our patients; thus, we were unable to demonstrate LVH regression in the study group. The intervention period was relatively short, and some subjects in the study group still had a higher nECW than the target level, and had HTN even though BP improved. Tian et al. also reported that BIA assistant intervention does not change a 1-year survival rate in PD [46]; therefore, it is difficult to conclude that BIA assistant dry-weight setting can provide a long-term survival benefit. Lastly, fluid control in the study group was lost in the maintenance phase, which makes the long-term effect of strict dry-weight control on RRF unviable. Despite these limitations, our work has several advantages compared to previous studies. First, peritoneal-membrane characteristics affecting ECW/TBW were illustrated, suggesting a complex interaction between peritoneal membrane and TBW composition. Second, unlike cross-sectional observational studies [28,34] that only give reference values, this study proved dry-weight intervention incorporating BIA measurement. An objective technique in dry-weight assessment was used, and good fluid status control that improved hemodynamics in patients on PD was demonstrated. Third, a proven accurate eight-polar BIA set was used as a warning target to avoid dehydration and control the rate of fluid reduction; this method efficiently reduces nECW and BP without accelerating RRF loss. These data show to nephrologists that patients with PD can achieve better fluid control without compromising the RRF to avoid dehydration.

In conclusion, this study revealed that peritoneal-membrane characteristics affect bodily-fluid composition, ECW/TBW ratio, dialysate protein loss, and protein malnutrition in PD. In addition, BIA is a useful guide to identify patients who are overhydrated, and a helpful objective technique for dry-weight assessment. For patients on PD who are hypervolemic, the correction of fluid overload improves BP control without adverse effects on RRF.

## Figures and Tables

**Table 1:** Demographic data categorized by blood pressure.

Variables	Normotensive PD	HTN-PD	Poor Controlled HTN PD	All Patient
Patient number	35	67	20	122
Age (years)	56.4 ± 14.5	56.3 ± 13.3	47.9 ± 12.0	55.1 ± 13.8
Gender (males)	12 (34%)	30 (45%)	9 (45%)	51 (41%)
Body weight (Kg)	55.7 ± 11.5	59.2 ± 11.2	62.2 ± 11.7	58.6 ± 11.5
DM	6 (17%)	14 (21%)	6 (30%)	26
SBP (mmHg)	114 ± 11	137 ± 13	165 ± 22	133 ± 21
DBP (mmHg)	70.1± 9.7	79.9 ± 11.0	95.2 ± 11.6	78.9 ± 13.2
HTN pill number	0	1.46	2.85	1.3
Albumin (g/dL)	3.61 ± 0.45	3.66 ± 0.55	3.68 ± 0.41	3.65 ± 0.50
Hemoglobin (g/dL)	10.1 ± 1.6	10 ± 1.5	9.6 ± 1.2	10 ± 1.5
D/P ratio	0.66 ± 0.10	0.65 ± 0.12	0.68 ± 0.08	0.66 ± 0.11
Kt/V	1.8 ± 0.43	1.74 ± 0.58	1.79 ± 0.49	1.77 ± 0.56
Urine kt/V	0.426 ± 0.51	0.366 ± 0.48	0.282 ± 0.29	0.37 ± 0.47
Urine volume (mL)	488 ± 557	512 ± 730	417 ± 463	492 ± 635

Abbreviations: DM, diabetes mellitus; SBP, systolic blood pressure; DBP, diastolic blood pressure.

**Table 2:** Demographic data after randomization.

Variables	Study Group	Control Group	*p*
Age	56.4 (25~80)	55.6 (20~83)	NS
Gender (males)	24 (40%)	27(43%)	NS
DM	14 (23%)	12 (19%)	NS
APD	4 (7%)	6 (10%)	NS
Weight (kg)	58.2 ± 1.4	58.1 ± 1.7	NS
BMI (Kg/M^2^ )	22.8 ± 4.0	22.5 ± 4.1	NS
Peritoneal D/P ratio	0.65 ± 0.13	0.66 ± 0.11	NS
Dialysis adequacy (nKT/V)	1.74 ± 0.08	1.77 ± 0.07	NS
ACEI/ARB prescription	16(26%)	18(30% )	NS
4.25% hypertonic PD solution	2(3%)	2(3%)	NS
Hematocrit (%)	29.8 ± 0.65	29.1 ± 0.52	NS
Total cholesterol (mg/dL)	182.6 ± 6.5	191.7 ± 5.8	NS
Serum BUN (mg/dL)	58.7 ± 2.7	58.5 ± 2.1	NS
Serum creatinine (mg/dL)	10.7 ± 0.4	10.7 ± 0.4	NS
ECW: TBW (×100%)	34.49 ± 1.00	34.76 ± 1.21	NS
nECW in females (L/m)	7.06 ± 0.95	6.96 ± 0.92	NS
nECW in males (L/m)	8.62 ± 0.99	8.89 ± 1.44	NS

Abbreviations: DM, diabetes mellitus; BMI, body-mass index; ECW, extracellular water; TBW, total body water; nECW, height normalized extracellular water.

**Table 3:** Changes in fluid status, hemodynamics, and residual renal function.

Data in the Beginning	Study Group (n = 60)	Control Group (n = 62)	*p*
Furosemide dosage (mg)	92.8 ± 10.2	85.2 ± 13.1	NS
Urine volume (ml/day)	888 ± 136 (n = 34)	698 ± 75 (n = 35)	NS
Renal KT/V	0.667 ± 0.085	0.587 ± 0.619	NS
Renal nWCC (L/week)	34.5 ± 4.4	30.6 ± 5.5	NS
SBP (mmHg)	132.4 ± 2.7	135.2 ± 3.3	NS
DBP (mmHg)	78.7 ± 1.7	78.2 ± 1.8	NS
Antihypertensive drugs	2.97 ± 0.77 (n = 32)	2.63 ± 0.75 (n = 29)	NS
ACEI/ARB prescription	16(31%)	18(36%)	NS
ECW: TBW (×100%)	34.49 ± 1.00	34.76 ± 1.21	NS
nECW in females (L/m)	7.06 ± 0.95	6.96 ± 0.92	NS
nECW in males (L/m)	8.62 ± 0.99	8.89 ± 1.44	NS
Albumin (g/dL)	3.73 ± 0.43	3.59 ± 0.53	NS
HS-CRP (md/dL)	0.430 ± 0.085	0.485 ± 0.104	NS
1.5% PD dialysate utilization (%)	84.6 ± 20.2	85.8 ± 19.2	NS
2.5% PD dialysate utilization (%)	23.2 ± 35.5	23.6 ± 35.3	NS
4.25% or ixoderin utilization (%)	1.9 ± 6.6	0.4 ± 3.2	NS
**End of the Intervention (6th M)**	**Study Group (n = 57)**	**Control Group (n = 55)**	
Delta body weight (kg)	−1.2 ± 0.4	0.1 ± 0.4	0.014
Furosemide dosage	96 ± 12.1	104.4 ± 13	NS
Urine volume (ml/day)	747 ± 150 (n= 31)	534 ± 84 (n= 31)	NS
Renal KT/V	0.52 ± 0.09	0.45 ± 0.09	NS
Renal nWCC (L/week)	24.9 ± 4.4	15.7 ± 3.2	NS
Patient with RRF loss 30%	4 (13%)	5 (16%)	NS
SBP (mmHg)	124.7 ± 2.7	136.8 ± 2.8	<0.001
DBP (mmHg)	74.9 ± 1.9	80.5 ± 2.0	0.050
Antihypertensive drugs	2.56 ± 0.40 (n = 31)	2.58 ± 0.30 (n = 29)	NS
ACEI/ARB prescription	13 (25%)	16 (32%)	NS
Delta ECW/TBW (×100%)	−0.063 ± 0.71	−0.059 ± 0.67	NS
Delta nECW (L/m)	−0.41 ± 0.13	−0.08 ± 0.07	0.042
Albumin (g/dL)	3.77 ± 0.075	3.57 ± 0.070	NS
HS-CRP (mg/dL)	0.520 ± 0.114	0.620 ± 0.093	NS
1.5% PD dialysate utilization (%)	81.3 ± 20.5	81.4 ± 19.3	NS
2.5% PD dialysate utilization (%)	28.5 ± 36.8	28.9 ± 35.6	NS
4.25% or ixoderin utilization (%)	2.1 ± 6.8	1.3 ± 5.7	NS
**End of the Maintenance (12th M)**	**Study Group (n = 52)**	**Control Group (n = 50)**	
Delta body weight	−0.78 ± 0.53	0.28 ± 0.49	NS
Furosemide dosage	93.2 ± 11.8	98.4 ± 12.9	NS
Urine volume (ml/day)	652 ± 174 (n = 25)	468 ± 112 (n = 26)	NS
Renal KT/V	0.42 ± 0.10	0.28 ± 0.06	NS
Renal nWCC (L/week)	21.8 ± 5.0	13.6 ± 3.3	NS
Patient with RRF loss >30%	8 (32%)	7 (27%)	NS
SBP (mmHg)	130.1 ± 3.2	136.5 ± 3.5	NS
DBP (mmHg)	77.4 ± 2.1	79.6 ± 2.1	NS
Antihypertensive drugs	2.95 ± 0.35 (n = 29)	2.77 ± 0.29 (n = 26)	NS
ACEI/ARB prescription	13 (25%)	14 (28%)	NS
Delta ECW/TBW (×100%)	−0.05 ± 0.89	−0.08 ± 0.70	NS
Delta nECW (L/m)	−0.03 ± 0.07	0.01 ± 0.12	NS
Albumin (g/dL)	3.65 ± 0.077	3.54 ± 0.89	NS
HS-CRP (mg/dL)	0.542 ± 0.102	0.811 ± 0.185	NS
1.5% PD dialysate utilization (%)	76.1 ± 18.9	76.0 ± 18.2	NS
2.5% PD dialysate utilization (%)	34.7 ± 35.9	35.7 ± 34.5	NS
4.25% or ixoderin utilization (%)	3.2 ± 8.4	2.5 ± 7.6	NS

## Data Availability

Not applicable.
